# Supplementary material for: Evidence for ethnic inequalities in mortality related to COVID-19 infections: findings from an ecological analysis of England
Source: BMJ Open. 2020 Dec 10;10(12):e041750. doi: 10.1136/bmjopen-2020-041750 (PMC7733217; doi:10.1136/bmjopen-2020-041750)
Supplement: Author's manuscript [file bmjopen-2020-041750.draft_revisions.pdf]

BMJ Open is committed to open peer review. As part of this commitment we make the peer review history of every article we publish publicly available.

When an article is published we post the peer reviewers' comments and the authors' responses online. We also post the versions of the paper that were used during peer review. These are the versions that the peer review comments apply to.

The versions of the paper that follow are the versions that were submitted during the peer review process. They are not the versions of record or the final published versions. They should not be cited or distributed as the published version of this manuscript.

BMJ Open is an open access journal and the full, final, typeset and author-corrected version of record of the manuscript is available on our site with no access controls, subscription charges or pay-per-view fees (http://bmjopen.bmj.com).

If you have any questions on BMJ Open's open peer review process please email info.bmjopen@bmj.com

BMJ Open

Evidence for ethnic inequalities in mortality related to COVID-19 infections: Findings from an ecological analysis of England and Wales

Journal:	*BMJ Open*
Manuscript ID	bmjopen-2020-041750
Article Type:	Original research
Date Submitted by the Author:	17-Jun-2020
Complete List of Authors:	Nazroo, James; The University of Manchester, Sociology<br>Becares, Laia; University of Sussex, Social Work and Social Care
Keywords:	EPIDEMIOLOGY, Epidemiology < INFECTIOUS DISEASES, Public health < INFECTIOUS DISEASES, PUBLIC HEALTH

SCHOLARONE™
Manuscripts

I, the Submitting Author has the right to grant and does grant on behalf of all authors of the Work (as defined in the below author licence), an exclusive licence and/or a non-exclusive licence for contributions from authors who are: i) UK Crown employees; ii) where BMJ has agreed a CC-BY licence shall apply, and/or iii) in accordance with the terms applicable for US Federal Government officers or employees acting as part of their official duties; on a worldwide, perpetual, irrevocable, royalty-free basis to BMJ Publishing Group Ltd ("BMJ") its licensees and where the relevant Journal is co-owned by BMJ to the co-owners of the Journal, to publish the Work in this journal and any other BMJ products and to exploit all rights, as set out in our licence.

The Submitting Author accepts and understands that any supply made under these terms is made by BMJ to the Submitting Author unless you are acting as an employee on behalf of your employer or a postgraduate student of an affiliated institution which is paying any applicable article publishing charge ("APC") for Open Access articles. Where the Submitting Author wishes to make the Work available on an Open Access basis (and intends to pay the relevant APC), the terms of reuse of such Open Access shall be governed by a Creative Commons licence – details of these licences and which Creative Commons licence will apply to this Work are set out in our licence referred to above.

Other than as permitted in any relevant BMJ Author's Self Archiving Policies, I confirm this Work has not been accepted for publication elsewhere, is not being considered for publication elsewhere and does not duplicate material already published. I confirm all authors consent to publication of this Work and authorise the granting of this licence.

For peer review only

Evidence for ethnic inequalities in mortality related to COVID-19 infections: Findings from an ecological analysis of England and Wales

James Nazroo
Professor of Sociology
Centre on Dynamics of Ethnicity
University of Manchester
Humanities Building
Oxford Road
Manchester M13 9PL
Email: james.nazroo@manchester.ac.uk

Laia Bécares
Senior Lecturer in Applied Social Sciences
Social Work and Social Care
University of Sussex
Essex House, Falmer, Brighton, BN1 9QQ
Email: L.Becares@sussex.ac.uk

Contributorship statement

Both authors were involved in the design, interpretation and writing of the manuscript. Nazroo carried out the analysis. The final manuscript was approved by both authors.

For peer review only - http://bmjopen.bmj.com/site/about/guidelines.xhtml

Abstract

Objectives

In the absence of robust direct data on ethnic inequalities in COVID-19 related mortality in the UK, we examine the relationship between ethnic composition of an area and rate of mortality in the area.

Design

Ecological analysis of COVID-19 related mortality rates occurring by 24th April 2020, and ethnic composition of the population. Account is taken of age, population density, area deprivation and pollution.

Setting

Local authorities in England and Wales.

Results

For every 1% rise in proportion of the population who are ethnic minority, COVID-19 related deaths increased by 5·10 (3·99 to 6·21) per million. This rise is present for each ethnic minority category examined, including the White minority group. The size of this increase is a little reduced in a fully adjusted model, suggesting that some of the association results from ethnic minority people living in more densely populated, more polluted and more deprived areas.

This estimate suggests that the average England and Wales COVID-19 related death rate would rise by 25% in a local authority with twice the average number of ethnic minority people.

Conclusions

We find clear evidence that rates of COVID-19 related mortality within a local authority increase as the proportion of the population who are ethnic minority increases. We suggest that this is a consequence of social and economic inequalities, including among key workers, driven by entrenched structural and institutional racism and racial discrimination. We argue that these factors should be central to any investigation of ethnic inequalities in COVID-19 outcomes.

Strengths and Limitations

- The analysis includes a comprehensive and recent count of deaths, including those occurring outside of clinical settings.
- The situation for different categories of ethnic minority group, including a white minority group, is examined.
- Robust and up to date denominators for the ethnic minority population are used, rather than relying on out of date or inaccurate estimates.
- Adjustments have been made for other factors that might explain the association between ethnicity and risk, including those that have been shown to be associated with increased mortality from COVID-19, such as pollution, or that have been found to be risk factors for mortality at the neighbourhood-level, such as deprivation and population density.
- However, these analyses are ecological, so inferences cannot be drawn about individuals from these aggregated data and the increased risk of mortality identified may be shared to a certain extent among different ethnic groups in an area.

For peer review only

For peer review only - http://bmjopen.bmj.com/site/about/guidelines.xhtml

Introduction

There is a growing body of evidence suggesting that there are marked ethnic inequalities in COVID-19 related deaths. Although UK data on ethnicity in relation to COVID-19 are sparse (as are data on ethnicity and health more generally), a report by The Intensive Care National Audit and Research Centre showed that around 35% of COVID-19 related admissions to intensive care were ethnic minority people and ethnic minority admissions were slightly more like to die in critical care (for example, 48·4% of White patients died in critical care compared with 55·3% of ethnic minority patients).[1] Further evidence published by the Guardian and Times newspapers suggested that ethnic minority people represent 19% of deaths recorded in hospital,[2] and that areas with a higher proportion of non-white ethnic minority people had higher death rates,[2] while, recent analysis of 106 healthcare workers who have died from covid-19 showed that 63% were from an ethnic minority background, and just over half were not born in the UK.[3] Given that non-white ethnic minority people made up 14% of the population in England and Wales at the 2011 Census, this suggests a marked inequality. This impression was reinforced by analysis of data released by the NHS, which suggested meaningful increases in death rates for ethnic minority people after taking into account differences in age structures and place of residence.[4] Evidence of ethnic/race inequalities in relation to COVID-19 has also been reported in the United States, where regional analyses indicate that areas with larger ethnic minority populations have higher rates of both COVID-19 infections and related deaths.[5] For example, in Michigan, a state where 15% of the population is Black, 40% of deaths are of Black people.[6]

Despite this accumulating evidence, it is hard to draw firm conclusions on the extent of ethnic inequalities in COVID-19 related risks. A large proportion of the evidence discussed in media reports is impressionistic, and where statistics on admissions or deaths are collated denominators are often missing, or are crudely estimated at a national level, rather than estimated from the population from which deaths are counted. In addition, the national data used to calculate denominators are typically drawn from the 2011 Census, which is considerably out of date and almost certainly underestimates the size of the ethnic minority population. Importantly, adjustments are rarely made for the younger age profile of ethnic minority people, nor for the potentially increased exposure to COVID-19 infection that results from their greater concentration in areas with a high population density, or greater risk from infection associated with higher levels of pollution. In addition, the data published rarely differentiate between different ethnic groups, and largely ignore White minority groups, meaning that potential differences between different ethnic minority groups are missed. Finally, the count of deaths often only includes those occurring in a hospitals, disregarding those who died in community settings.

To begin to address these issues and to enhance our understanding of ethnic inequalities in COVID-19 related risks, we conduct an ecological analysis, at local authority level, of the relationship between COVID-19 related deaths and the proportion of the local population who are ethnic minority. To do this we use the most recent release of data, accurate estimates of the size and ethnic composition of the population in local authorities, and a modelling approach that accounts for some potential explanations for the higher risk faced by ethnic minority people.

Methods

Death rates are taken from ONS 5th May 2020 release of deaths in England and Wales, by local authority.[7] (Local authorities are government organisations officially responsible for all the public services and facilities for both individuals and business in an area.) This release includes all deaths, including those that occurred in community settings, that occurred up to 24th April and that were

For peer review only - http://bmjopen.bmj.com/site/about/guidelines.xhtml

registered by the 2nd May. Only those deaths recorded as involving COVID-19, a recording that is based on any mention of COVID-19 on the death certificate, are included. These figures do not include deaths of those who reside outside England and Wales, or deaths where the place of residence is either missing or not yet fully coded on the death certificate. There were 27,287 COVID-19 related deaths in England and Wales by 24th April, as recorded on 2nd May.

To provide the denominator to calculate COVID-19 related death rates by local authority the ONS 2018 mid-year population estimates are used.[8] These data estimate the England and Wales population as 59,115,809, meaning that the COVID-19 related death rate for England and Wales was 46·16 per 100,000 as of 24th April 2020.

To examine the association between COVID-19 related deaths and proportion of the local population who are non-white ethnic minority across local authorities, we use linear regression models with COVID-19 related death rate as the dependent variable. The main predictor variable, the proportion of the population who are ethnic minority, was taken from the ETHPOP estimates for 2018 (we used the 2018 used rather than those for 2020 in order to match in time with the ONS population estimates used for total population size).[9] These estimates are based on sophisticated modelling of change in the ethnic composition of the population at local authority level, accounting for births, deaths and migration both into the UK and within the UK. This is done separately for different ethnic groups, the categories used being: White British/Irish/Traveller; Indian; Pakistani; Bangladeshi; Chinese; Other Asian; Black African; Black Caribbean; Other Black; Mixed; White Other; and Other. For 2018 this model estimates that the England and Wales population was 77% White British/Irish/Traveller. Given the relatively small population sizes of ethnic minority groups and their concentration in particular regions of England and Wales, the analysis conducted here combine ethnic minority groups into four categories: Asian (Indian, Pakistani, Bangladeshi, Chinese, Other Asian); Black (Black African, Black Caribbean, Other Black); White Other; and Other (Mixed, Other). For the more detailed modelling all of the ethnic minority groups are combined together and compared with the White British/Irish/Traveller group. In each case, these variables are modelled as a continuous variable that captures a 1% increase in the concentration of the group in a local authority.

The following covariates are included in the analysis: percentage of the population who were aged 70 or over (using the ONS 2018 mid-year population estimates);[8] population density (number of people per square kilometre, using the ONS 2018 mid-year population estimates);[8] the 2019 Index of Multiple Deprivation score;[9] and levels of pollution as marked by population-weighted annual mean PM2.5 (anthropogenic) concentration for 2018 (ugm-3).[11]

We use linear regression models to predict variation in COVID-19 related death rate across local authorities. First simple, descriptive models are used to show the association between each ethnic minority category and risk of death, and the association between each covariate and risk of death. Then we included all the predictor variables to show the fully-adjusted relationship between COVID-19 related deaths and the proportion of the local population who are ethnic minority.

Patient and Public Involvement

Patients or the public were not involved in the design, or conduct, or reporting, or dissemination plans of this research. The research was based entirely on secondary analysis of existing administrative data.

For peer review only - http://bmjopen.bmj.com/site/about/guidelines.xhtml

Results

Table 1 presents the results of the crude descriptive models, showing unadjusted coefficients for the relationship between proportion of the population in a local authority who are of an ethnic minority group and the COVID-19 related death rate. For ease of interpretation the coefficients have been estimated as the change in number of deaths per million for a 1% rise in proportion of the population who are in the ethnic group. Although descriptive in nature, this analysis indicates a marked inequality between areas, with a rise in the ethnic minority population in an area related to a statistically significant higher rate of COVID-19 related death. This is present for each of the ethnic categories shown in the table, and also present for each sub-category in a disaggregated analysis (not shown but available from the authors). Most confidence can be placed on the estimate for the total ethnic minority population, which combines all of the other groups, because the population size is relatively large and more evenly distributed across local authorities. This coefficient shows that for every 1% rise in proportion of the population who are ethnic minority COVID-19 related deaths increased by 5·10 (3·99 to 6·21) per million.

Table 1: Association between size of ethnic population and COVID-19 deaths at local authority level: England and Wales

				Death rate per million for 1% increase in population size
Asian	Black	White minority, excluding Irish and Travellers	Other incl· Mixed	Total ethnic minority
9·00<br>(6·75 – 11·25)	19·91<br>(15·38 – 24·43)	10·37<br>(6·15 – 14·59)	32·92<br>(25·47 – 40·37)	5·10<br>(3·99 – 6·21)

Table 2 presents a descriptive analysis of the relationship between covariates and COVID-19 related death rates in a local authority.

Table 2: Association between area characteristics and COVID-19 deaths at local authority level: England and Wales

1% increase in proportion of population aged 70 or older	Population density (100 per square km)	Index of Multiple Deprivation	Pollution
-0·21<br>(-0·27 – -0·16)	0·27<br>(0·20 – 0·35)	0·27<br>(0·034 – 0·50)	0·35<br>(0·25 – 0·45)

Each of these area characteristics are significantly associated with risk of death, with population density, Index of Multiple Deprivation and level of pollution all increasing risk. However, surprisingly, the proportion of the population who are aged 70 or older, a risk factor for COVID-19 related death, is negatively associated with risk of death, although the effect is very small. Subsequent analysis (not shown, but available from the authors) revealed that this was possibility a result of the negative correlation between the proportion of ethnic minority people and the proportion of people aged 70 and over in an area.

Finally, Table 3 presents the full model, with each of the risk factors present. Once all variables are included, only the proportion of the population who are ethnic minority remains statistically

For peer review only - http://bmjopen.bmj.com/site/about/guidelines.xhtml

significant. The estimated coefficient for proportion ethnic minority is a little reduced compared with the unadjusted figure, going from 5·10 (3·99 to 6·21) to 3·69 (1·57 to 5·82), suggesting that some of the ethnic minority association results from ethnic minority people living in more densely populated, more polluted and more deprived areas.

Table 3: Multivariate analysis of association between area characteristics and COVID-19 deaths at local authority level: England and Wales

	1% increase in proportion ethnic minority	1% increase in proportion aged 70 or older	Population density (100 per square km)	Index of Multiple Deprivation	Pollution
Death rate per million	3·69 (1·57 – 5·82)	-0·044	-0·0015	0·022	0·12 (-0·01 – 0·25)
Probability	<0·001	0·33	0·82	0·85	<0·1

To illustrate the significance of these findings, if we take the average England and Wales COVID-19 related death rate as 46·16 per hundred thousand, for a local authority with twice the average number of ethnic minority people (using 2018 ETHPOP estimates this would be an increase from 23% to 46%) would have a predicted COVID-19 related death rate of 57·89 per hundred thousand (46·16+(23*5·10/10)), an increase of 25%. While a local authority with a small proportion of ethnic minority people, say 3% rather than 23%, would have a predicted death rate of 35·96 per hundred thousand (46·16-(20*5·10/10)), a decrease of almost a quarter.

Discussion

The analyses presented here provide the strongest evidence to date on ethnic inequalities in COVID-19 related mortality in England and Wales. Findings from these analyses show a clear association between an increase in the proportion of ethnic minority residents in a local authority and an increase in mortality related to COVID-19. Of importance is that this analysis provides robust and up to date denominators for the ethnic minority population, a comprehensive and recent count of deaths, examines the situation for different categories of ethnic minority groups, and that it includes adjustments for other factors that might explain the association between ethnicity and risk, including those that have been shown to be associated with increased mortality from COVID-19, such as pollution,[12] or that have been found to be risk factors for mortality at the neighbourhood-level, such as deprivation,[13] and population density.[14]

However, it is important to bare in mind that these analyses are ecological, so inferences cannot be drawn about individuals from these aggregated data. This means that the increased risk of mortality identified may be shared to a certain extent among different ethnic groups in the area, although the evidence of an association between increased risk and each of the categories of ethnic minority group examined is suggestive of the risk not being equally shared between ethnic minority and ethnic majority people living in an area. In addition, we can draw from existing evidence documenting ethnic inequalities across a range of socioeconomic, mental health, and physical health outcomes,[15-17] including non-COVID-19 related mortality,[18] to support the likelihood that ethnic minority residents suffer from an increased risk of exposure and vulnerability to COVID-19 infection and related mortality, and whose increased risk, therefore, is likely to be a key factor driving these findings.

For peer review only - http://bmjopen.bmj.com/site/about/guidelines.xhtml

There has been much public debate about what might be driving apparent ethnic inequalities in risk of COVID-19 related complications and death. Most of these discussions point towards the role of underlying social and economic inequalities that are faced by ethnic minority people. That is, most ethnic minority groups are more vulnerable to, and have poorer prognosis from, COVID-19 infection, because they are more likely to: be poorer; have poorly paid and insecure employment; live in over-crowded housing; and live in deprived neighbourhoods with high rates of concentrated poverty and increased pollution levels.[19] The fully adjusted model, which shows a reduction in the coefficient related to proportion of the population who are ethnic minority once factors such as these are taken into account, provides some support for these possibilities. However, it is important to note again that these are ecological analyses, so do not account for the very likely possibility that ethnic minority people have poorer circumstances than others living in the same area.

Ethnic minority people are also more likely to be employed in sectors that increase their risk of exposure to COVID-19. An over-representation of ethnic minority people can be found working in transport and delivery jobs, as health care assistants, hospital cleaners, social care workers, and in nursing and medical jobs. Not only do these occupations increase risk of infection, some of these are also occupations that have been the last to receive supplies of personal protective equipment. People in these occupations have now been deemed key workers, but for decades ethnic minority people working in these jobs have endured job insecurity, low pay, and discrimination.

In addition, ethnic minority people are more likely to have the underlying health conditions that have been linked to increased risk of COVID-19 related complications and mortality, such as asthma, diabetes, high blood pressure, and coronary heart disease.[20] These health conditions are socially-patterned, so that the social and economic inequalities faced by ethnic minority people described above, lead to an increased risk of developing these health conditions.[15] As a result, we would argue that the increased risks associated with COVID-19 infection faced by ethnic minority people are now a core component of wider ethnic inequalities in health, and these negative consequences are amplified by long established pre-existing ethnic inequalities in health, both of which are driven by social and economic inequalities.

Behind this complexity, however, is a key consideration that is typically absent from investigations into ethnic inequalities in health - that the social and economic inequalities that are faced by ethnic minority people are driven by entrenched structural and institutional racism and racial discrimination.[21] An explanation of ethnic inequalities that stops at social and economic inequalities and doesn't acknowledge how these inequalities have been, and continue to be, shaped by historical processes underpinned by racism, will be limited in its ability to generate an understanding of, and solutions to, ethnic inequalities. A myriad of studies in the UK and elsewhere have now documented the role of racism in patterning inequalities in education, employment, income, housing, and proximity to pollution.[22-26] In addition, experiences of racial discrimination have been linked to a numerous mental and physical health outcomes, including asthma and hypertension.[17,21,27] Importantly, these processes do not operate in isolation, they co-occur and sequentially lead to deepening inequalities in many domains across a person's life course, and are transmitted from one generation to the next.[28,29]

Excluding racism – the root of ethnic inequalities in COVID-19 infections and related mortality – from scientific and policy discussions around the determinants and implications of the coronavirus pandemic can lead to dangerous and ineffective investigations and policy interventions. These include un-evidenced reductionist approaches which question whether ethnic inequalities in COVID-19 may be due to biological/genetic or cultural differences, a line of thinking that risks taking us back into a time of scientific racism, but which is, for example, reflected in a recent call for research on

For peer review only - http://bmjopen.bmj.com/site/about/guidelines.xhtml

this issue.[30] Before we respond to such an agenda we should ask ourselves the simple question: 'what could possibly be the biological or cultural similarities between an ethnic minority family living in Tower Hamlets, London and another living in Detroit, Michigan, both of whom face an increased risk of COVID-19 related complications and mortality?' More likely than shared genetic and cultural risks, is that they will both live in disinvested neighbourhoods with high levels of pollution and concentrated poverty, with insecure and underpaid employment, and in overcrowded conditions with substandard levels of housing. Chances are they have had their lives shaped by institutional and structural racism, and have experiences of racial discrimination deeply embedded in their lives. These are the similarities that policy and research efforts should be paying attention to. Given this, the increased risks faced by ethnic minority people should not have been unexpected, as appears to have been the case, they could and should have been anticipated.

That Public Health England is reviewing ethnic inequalities in COVID-19 related outcomes could be an important shift of focus, especially when contemporary policy work around inequalities in health have largely ignored the question of ethnicity.[31,32] However, it is crucial that this review considers how current inequalities relate to longstanding ethnic inequalities in health and in doing so the question of racism must not be side-stepped. Similarly, the review must also focus on the greater harm done to ethnic minority people as a result of Government responses to the coronavirus pandemic and move quickly to consider how these greater harms might be mitigated. The justification for the pandemic measures is that their estimated effect on reducing the impact of the COVID-19 pandemic on the NHS, by protecting its capacity to provide care for people who become seriously ill as a result of a COVID-19 infection, would offset their acknowledged extremely negative economic, social, health and psychological impacts. That is, the negative is on average judged to be worth the estimated direct health benefits. However, the situation facing ethnic minority people is far more precarious than 'the average', as detailed above, meaning that these measures are certainly having a more negative effect on ethnic minority people. In addition, some of the more punitive dimensions of 'lockdown', such as changes in the Mental Health Act,[33] police surveillance, and discontinuity in the clinical management of pre-existing conditions, are also going to more adversely impact those with racialised identities.

References

1. ICNARC report on COVID-19 in critical care 24 April 2020, https://www.icnarc.org/Our-Audit/Audits/Cmp/Reports, accessed 26th April
2. Ethnic minorities dying of Covid-19 at higher rate, analysis shows. The Guardian, 22 April 2020. Available at: https://www.theguardian.com/world/2020/apr/22/racial-inequality-in-britain-found-a-risk-factor-for-covid-19
3. Cook T, Kursumovic E, Lennane S. Exclusive: deaths of NHS staff from covid-19 analysed. Health Serv J 2020 Apr 22. https://www.hsj.co.uk/exclusive-deaths-of-nhs-staff-fromcovid-19-analysed/7027471.article
4. Platt, L. and Warwick R. (2020) Are some ethnic groups more vulnerable to COVID-19 than others? Institute for Fiscal Studies ISBN 978-1-912805-75-4
5. Thebault R, Tran AB, Williams V. The coronavirus is infecting and killing black Americans at an alarmingly high rate. Washington Post 2020 Apr 7. https://www.washingtonpost.com/nation/2020/04/07/coronavirus-is-infecting-killing-black-americans-an-alarmingly-high-rate-post-analysis-shows
6. https://www.propublica.org/article/early-data-shows-african-americans-have-contracted-and-died-of-coronavirus-at-an-alarming-rate
7. https://www.ons.gov.uk/peoplepopulationandcommunity/healthandsocialcare/causesofdeath/datasets/deathregistrationsandoccurrencesbylocalauthorityandhealthboard, accessed 28th April

For peer review only - http://bmjopen.bmj.com/site/about/guidelines.xhtml

8. https://www.ons.gov.uk/peoplepopulationandcommunity/populationandmigration/populationestimates/datasets/populationestimatesforukenglandandwalesscotlandandnorthernireland
9. Wohland P, Burkitt M, Norman P, Rees P, Boden P and Durham H, ETHPOP Database, ESRC Follow on Fund "Ethnic group population trends". www.ethpop.org. Date of extraction 26th April 2020.
10. https://assets.publishing.service.gov.uk/government/uploads/system/uploads/attachment_data/file/835115/IoD2019_Statistical_Release.pdf
11. https://uk-air.defra.gov.uk/data/pcm-data#population_weighted_annual_mean_pm25_data
12. Wu, X., Nethery, R., Sabath, B., Braun, D., & Dominici, F. (2020). Exposure to air pollution and COVID-19 mortality in the United States medRxiv 2020.04.05.20054502; doi: https://doi.org/10.1101/2020.04.05.20054502
13. Singh G. K. (2003). Area deprivation and widening inequalities in US mortality, 1969-1998. American journal of public health, 93(7), 1137–1143. https://doi.org/10.2105/ajph.93.7.1137
14. Beenackers, M., Groeniger, J., Kamphuis, C., Van Lenthe, F. (2018). Urban population density and mortality in a compact Dutch city: 23-year follow-up of the Dutch GLOBE study, Health & Place, 53, 79-85.
15. Nazroo J. Y. (2003). The structuring of ethnic inequalities in health: economic position, racial discrimination, and racism. American journal of public health, 93(2), 277–284. https://doi.org/10.2105/ajph.93.2.277
16. Bécares, L. (2015). Which ethnic groups have the poorest health?. In Jivraj, S. and Simpson, L. (eds.) Ethnic identity and inequalities in Britain. Bristol: The Policy Press
17. Wallace, S., Nazroo, J.Y. and Bécares, L. (2016)Cumulative exposure to racial discrimination across time and domains: exploring racism's long term impact on the mental health of ethnic minority people in the UK. American Journal of Public Health, 106(7), 1294-1300. doi: 10.2105/AJPH.2016.303121
18. Bos, V., Kunst, A., Keij-Deerenberg, I., Garssen, J., & Mackenbach, J. (2004). Ethnic inequalities in age- and cause-specific mortality in The Netherlands, International Journal of Epidemiology, 33(5), 1112–1119.
19. Byrne, B., Alexander, C., Khan, O., Nazroo, J. And Shankley, W. (eds.) (2020) Ethnicity, Race and Inequality in the UK: State of the Nation, Bristol: Policy Press ISBN 978-1447351269)
20. Nazroo, J.Y. (1997) The health of Britain's ethnic minorities: findings from a national survey, London: Policy Studies Institute ISBN 0 85374 709 1
21. Nazroo, J., Bhui, K. and Rhodes, J. (2020) 'Where next for understanding race/ethnic inequalities in severe mental illness? Structural, interpersonal and institutional racism', *Sociology of Health and Illness*, 42, 2, 262-276, doi: 10.1111/1467-9566.130012
22. Alexander, C. and Arday, J. (eds) (2015) Aiming Higher: Race Inequality and Diversity in the Academy.London: Runnymede Trust.
23. Zschirnt, E. and Ruedin, D. (2016): Ethnic discrimination in hiring decisions: a meta-analysis of correspondence tests 1990–2015. Journal of Ethnic and Migration Studies 42(7): 1115-34.
24. Ashe, S. and Nazroo, J. (2016) Equality, Diversity and Racism in the Workplace: A Qualitative Analysis of the 2015 Race at Work Survey, London: Business in The Community, https://race.bitc.org.uk/system/files/research/race_at_work_equality_diversity_and_racism_in_the_workplace_november_2016.pdf
25. Hogan, Bernie, and Brent Berry. 2011. "Racial and Ethnic Biases in Rental Housing: An Audit Study of Online Apartment Listings." City and Community 10 (4): 351–372.
26. Cohen, H. W., & Northridge, M. E. (2008). Getting political: racism and urban health. American journal of public health, 98(9 Suppl), S17–S19. https://doi.org/10.2105/ajph.98.supplement_1.s17
27. Bécares, L., Stafford, M., & Nazroo, J. (2009). Fear of racism, employment and expected organizational racism: their association with health. European Journal of Public Health, 19(5), 504-510.

For peer review only - http://bmjopen.bmj.com/site/about/guidelines.xhtml

28. Gee, G. C., Walsemann, K. M., & Brondolo, E. (2012). A life course perspective on how racism may be related to health inequities. American journal of public health, 102(5), 967–974. https://doi.org/10.2105/AJPH.2012.300666
29. Bécares, L., Nazroo, J., & Kelly, Y. (2015). A longitudinal examination of maternal, family, and area-level experiences of racism on children's socioemotional development: patterns and possible explanations. Social Science & Medicine, 142, 128-135
30. NIHR and UKRI Launch call for research on COVID-19 and ethnicity. Available at: https://www.nihr.ac.uk/news/nihr-and-ukri-launch-call-for-research-on-covid-19-and-ethnicity/24658
31. https://www.bmj.com/content/368/bmj.m268.full%22
32. Salway, S., Holman, D., Lee, C., McGowan, V., Ben-Shlomo, Y., Saxena, S. and Nazroo, J. (2020) 'Transforming the health system for the UK's multi-ethnic population', British Medical Journal, 368, m268 doi: 10.1136/bmj.m268
33. https://synergicollaborativecentre.co.uk/new-coronavirus-act-may-increase-the-mental-health-detention-of-ethnic-minority-people/%22

Acknowledgements
We are grateful for the support provided by Caelainn Barr, Editor, Data projects, Guardian News and Media, and Niamh McIntyre, Niko Kommenda and Antonio Voce, Data projects, Guardian News and Media.

Funding
Nazroo's work on this paper was funded by the Economic and Social Research Council, grant number ES/R009341/1.

Data sharing
The analyses presented here make use of publicly available data. Links to the data are provided within the paper.

For peer review only

STROBE Statement—Checklist of items that should be included in reports of ***cross-sectional studies***

	Item No	Recommendation	Page No
**Title and abstract**	1	(*a*) Indicate the study's design with a commonly used term in the title or the abstract	1
		(*b*) Provide in the abstract an informative and balanced summary of what was done and what was found	2
**Introduction**			
Background/rationale	2	Explain the scientific background and rationale for the investigation being reported	3
Objectives	3	State specific objectives, including any prespecified hypotheses	3
**Methods**			
Study design	4	Present key elements of study design early in the paper	3-4
Setting	5	Describe the setting, locations, and relevant dates, including periods of recruitment, exposure, follow-up, and data collection	3-4
Participants	6	(*a*) Give the eligibility criteria, and the sources and methods of selection of participants	N/a
Variables	7	Clearly define all outcomes, exposures, predictors, potential confounders, and effect modifiers. Give diagnostic criteria, if applicable	4
Data sources/ measurement	8*	For each variable of interest, give sources of data and details of methods of assessment (measurement). Describe comparability of assessment methods if there is more than one group	*4*
Bias	9	Describe any efforts to address potential sources of bias	N/a
Study size	10	Explain how the study size was arrived at	N/a
Quantitative variables	11	Explain how quantitative variables were handled in the analyses. If applicable, describe which groupings were chosen and why	4
Statistical methods	12	(*a*) Describe all statistical methods, including those used to control for confounding	4
		(*b*) Describe any methods used to examine subgroups and interactions	4
		(*c*) Explain how missing data were addressed	N/a
		(*d*) If applicable, describe analytical methods taking account of sampling strategy	4
		(*e*) Describe any sensitivity analyses	N/a
**Results**			
Participants	13*	(a) Report numbers of individuals at each stage of study—eg numbers potentially eligible, examined for eligibility, confirmed eligible, included in the study, completing follow-up, and analysed	N/a
		(b) Give reasons for non-participation at each stage	N/a
		(c) Consider use of a flow diagram	N/a
Descriptive data	14*	(a) Give characteristics of study participants (eg demographic, clinical, social) and information on exposures and potential confounders	N/a
		(b) Indicate number of participants with missing data for each variable of interest	N/a
Outcome data	15*	Report numbers of outcome events or summary measures	4
Main results	16	(*a*) Give unadjusted estimates and, if applicable, confounder-adjusted estimates and their precision (eg, 95% confidence interval). Make clear which confounders were adjusted for and why they were included	5

For peer review only

		(*b*) Report category boundaries when continuous variables were categorized	N/a
		(*c*) If relevant, consider translating estimates of relative risk into absolute risk for a meaningful time period	N/a
Other analyses	17	Report other analyses done—eg analyses of subgroups and interactions, and sensitivity analyses	5
**Discussion**			
Key results	18	Summarise key results with reference to study objectives	6
Limitations	19	Discuss limitations of the study, taking into account sources of potential bias or imprecision. Discuss both direction and magnitude of any potential bias	6
Interpretation	20	Give a cautious overall interpretation of results considering objectives, limitations, multiplicity of analyses, results from similar studies, and other relevant evidence	6-8
Generalisability	21	Discuss the generalisability (external validity) of the study results	N/a
**Other information**			
Funding	22	Give the source of funding and the role of the funders for the present study and, if applicable, for the original study on which the present article is based	1

*Give information separately for exposed and unexposed groups.

Note: An Explanation and Elaboration article discusses each checklist item and gives methodological background and published examples of transparent reporting. The STROBE checklist is best used in conjunction with this article (freely available on the Web sites of PLoS Medicine at http://www.plosmedicine.org/, Annals of Internal Medicine at http://www.annals.org/, and Epidemiology at http://www.epidem.com/). Information on the STROBE Initiative is available at www.strobe-statement.org.

For peer review only

For peer review only - http://bmjopen.bmj.com/site/about/guidelines.xhtml

BMJ Open

Evidence for ethnic inequalities in mortality related to COVID-19 infections: Findings from an ecological analysis of England

Journal:	*BMJ Open*
Manuscript ID	bmjopen-2020-041750.R1
Article Type:	Original research
Date Submitted by the Author:	01-Sep-2020
Complete List of Authors:	Nazroo, James; The University of Manchester, Sociology<br>Becares, Laia; University of Sussex, Social Work and Social Care
**Primary Subject Heading**:	Epidemiology
Secondary Subject Heading:	Public health, Sociology
Keywords:	EPIDEMIOLOGY, Epidemiology < INFECTIOUS DISEASES, Public health < INFECTIOUS DISEASES, PUBLIC HEALTH

SCHOLARONE™
Manuscripts

I, the Submitting Author has the right to grant and does grant on behalf of all authors of the Work (as defined in the below author licence), an exclusive licence and/or a non-exclusive licence for contributions from authors who are: i) UK Crown employees; ii) where BMJ has agreed a CC-BY licence shall apply, and/or iii) in accordance with the terms applicable for US Federal Government officers or employees acting as part of their official duties; on a worldwide, perpetual, irrevocable, royalty-free basis to BMJ Publishing Group Ltd ("BMJ") its licensees and where the relevant Journal is co-owned by BMJ to the co-owners of the Journal, to publish the Work in this journal and any other BMJ products and to exploit all rights, as set out in our licence.

The Submitting Author accepts and understands that any supply made under these terms is made by BMJ to the Submitting Author unless you are acting as an employee on behalf of your employer or a postgraduate student of an affiliated institution which is paying any applicable article publishing charge ("APC") for Open Access articles. Where the Submitting Author wishes to make the Work available on an Open Access basis (and intends to pay the relevant APC), the terms of reuse of such Open Access shall be governed by a Creative Commons licence – details of these licences and which Creative Commons licence will apply to this Work are set out in our licence referred to above.

Other than as permitted in any relevant BMJ Author's Self Archiving Policies, I confirm this Work has not been accepted for publication elsewhere, is not being considered for publication elsewhere and does not duplicate material already published. I confirm all authors consent to publication of this Work and authorise the granting of this licence.

For peer review only

Evidence for ethnic inequalities in mortality related to COVID-19 infections: Findings from an ecological analysis of England

James Nazroo (corresponding author)
Professor of Sociology
Centre on Dynamics of Ethnicity
University of Manchester
Humanities Building
Oxford Road
Manchester M13 9PL
Email: james.nazroo@manchester.ac.uk

Laia Bécares
Senior Lecturer in Applied Social Sciences
Social Work and Social Care
University of Sussex
Essex House, Falmer, Brighton, BN1 9QQ
Email: L.Becares@sussex.ac.uk

For peer review only

Contributorship statement

J. Nazroo and L. were both involved in the design of the study, interpretation of the findings, and writing of the manuscript. J. Nazroo carried out the analysis. The final manuscript was approved by both J. Nazroo and L. Bécares.

Competing interests

There are no competing interests for any author.

For peer review only - http://bmjopen.bmj.com/site/about/guidelines.xhtml

Abstract

Objectives
In the absence of robust direct data on ethnic inequalities in COVID-19 related mortality in the UK, we examine the relationship between ethnic composition of an area and rate of mortality in the area.

Design
Ecological analysis of COVID-19 related mortality rates occurring by 24th April 2020, and ethnic composition of the population. Account is taken of age, population density, area deprivation and pollution.

Setting
Local authorities in England.

Results
For every 1% rise in proportion of the population who are ethnic minority, COVID-19 related deaths increased by 5·12 (4·00 to 6·24) per million. This rise is present for each ethnic minority category examined, including the White minority group. The size of this increase is a little reduced in an adjusted model to 4·42 (2·24 to 6·60), suggesting that some of the association results from ethnic minority people living in more densely populated, more polluted and more deprived areas.

This estimate suggests that the average England COVID-19 related death rate would rise by 25% in a local authority with twice the average number of ethnic minority people.

Conclusions
We find clear evidence that rates of COVID-19 related mortality within a local authority increases as the proportion of the population who are ethnic minority increases. We suggest that this is a consequence of social and economic inequalities, including among key workers, driven by entrenched structural and institutional racism and racial discrimination. We argue that these factors should be central to any investigation of ethnic inequalities in COVID-19 outcomes.

Strengths and Limitations

- The analysis includes a comprehensive and recent count of deaths, including those occurring outside of clinical settings.
- The situation for different categories of ethnic minority group, including a white minority group, is examined.
- Robust and up to date denominators for the ethnic minority population are used, rather than relying on out of date or inaccurate estimates.
- Partial adjustments have been made for other factors that might explain the association between ethnicity and risk, including those that have been shown to be associated with increased mortality from COVID-19, such as pollution, or that have been found to be risk factors for mortality at the neighbourhood-level, such as deprivation and population density.
- However, these analyses are ecological, so inferences cannot be drawn about individuals from these aggregated data and the increased risk of mortality identified may be shared to a certain extent among different ethnic groups in an area.

For peer review only

For peer review only - http://bmjopen.bmj.com/site/about/guidelines.xhtml

Introduction

There is a growing body of evidence suggesting that there are marked ethnic inequalities in COVID-19 related deaths. Although UK data on ethnicity in relation to COVID-19 are sparse (as are data on ethnicity and health more generally), a report by The Intensive Care National Audit and Research Centre showed that around 35% of COVID-19 related admissions to intensive care were ethnic minority people and ethnic minority admissions were slightly more like to die in critical care (for example, 48·4% of White patients died in critical care compared with 55·3% of ethnic minority patients).[1] Further evidence published by the Guardian and Times newspapers, based on their own, non-peer reviewed research, suggested that ethnic minority people represent 19% of deaths recorded in hospital,[2] and that areas with a higher proportion of non-white ethnic minority people had higher death rates,[2] while, recent analysis of 106 healthcare workers who have died from covid-19 showed that 63% were from an ethnic minority background, and just over half were not born in the UK.[3] Given that non-white ethnic minority people made up 14% of the population in England and Wales at the 2011 Census, this suggests a marked inequality. This impression was reinforced by secondary analysis of data released by the NHS, which suggested meaningful increases in death rates for ethnic minority people after taking into account differences in age structures and place of residence.[4] Evidence of ethnic/race inequalities in relation to COVID-19 has also been reported in the United States, where regional analyses indicate that areas with larger ethnic minority populations have higher rates of both COVID-19 infections and related deaths.[5] For example, in Michigan, a state where 15% of the population is Black, 40% of deaths are of Black people.[6]

Despite this accumulating evidence, it is hard to draw firm conclusions on the extent of ethnic inequalities in COVID-19 related risks. A large proportion of the evidence discussed in media reports is impressionistic, and where statistics on admissions or deaths are collated denominators are often missing, or are crudely estimated at a national level, rather than estimated from the population from which deaths are counted. In addition, the national data used to calculate denominators are typically drawn from the 2011 Census, which is considerably out of date and almost certainly underestimates the size of the ethnic minority population. Importantly, adjustments are rarely made for the younger age profile of ethnic minority people, nor for the potentially increased exposure to COVID-19 infection that results from their greater concentration in areas with a high population density, or greater risk from infection associated with higher levels of pollution and living in areas with greater levels of deprivation. In addition, the data published rarely differentiate between different ethnic groups, and largely ignore White minority groups, meaning that potential differences between different ethnic minority groups are missed. Finally, the count of deaths often only includes those occurring in a hospitals, disregarding those who died in community settings.

To begin to address these issues and to enhance our understanding of ethnic inequalities in COVID-19 related risks, we conduct an ecological analysis, at local authority level, of the relationship between COVID-19 related deaths and the proportion of the local population who are ethnic minority. To do this we use the most recent release of data at the time of writing, accurate estimates of the size and ethnic composition of the population in local authorities, and a modelling approach that accounts for some potential explanations, noted above, for the higher risk faced by ethnic minority people.

Methods

Death rates are taken from ONS 5th May 2020 release of deaths in England, by local authority.[7] (Local authorities are government organisations officially responsible for all the public services and facilities for both individuals and business in an area.) This release includes all deaths, including those that occurred in community settings, that occurred up to 24th April and that were registered by the 2nd

For peer review only - http://bmjopen.bmj.com/site/about/guidelines.xhtml

May. Only those deaths recorded as involving COVID-19, a recording that is based on any mention of COVID-19 on the death certificate, are included. These figures do not include deaths of those who reside outside England, or deaths where the place of residence is either missing or not yet fully coded on the death certificate. There were 26,004 COVID-19 related deaths in England by 24th April, as recorded on 2nd May.

To provide the denominator to calculate COVID-19 related death rates by local authority the ONS 2018 mid-year population estimates are used.[8] These data estimate the England population as 55,977,178, meaning that the COVID-19 related death rate for England was 46·45 per 100,000 as of 24th April 2020.

To examine the association between COVID-19 related deaths and proportion of the local population who are ethnic minority across local authorities, we use linear regression models with COVID-19 related death rate as the dependent variable. The main predictor variable, the proportion of the population who are ethnic minority, was taken from the ETHPOP estimates for 2018 (we used the 2018 used rather than those for 2020 in order to match in time with the ONS population estimates used for total population size).[9] These estimates are based on sophisticated modelling of change in the ethnic composition of the population at local authority level, accounting for births, deaths and migration both into the UK and within the UK. This is done separately for different ethnic groups, the categories used being: White British/Irish/Traveller; Indian; Pakistani; Bangladeshi; Chinese; Other Asian; Black African; Black Caribbean; Other Black; Mixed; White Other; and Other. For 2018 this model estimates that the England population was 77.2% White British/Irish/Traveller. Given the relatively small population sizes of ethnic minority groups and their concentration in particular regions of England, the analysis conducted here combine ethnic minority groups into four categories: Asian (Indian, Pakistani, Bangladeshi, Chinese, Other Asian); Black (Black African, Black Caribbean, Other Black); White Other; and Other (Mixed, Other). For the more detailed modelling all of the ethnic minority groups are combined together and compared with the White British/Irish/Traveller group. In each case, these variables are modelled as a continuous variable that captures a 1% increase in the concentration of the group in a local authority.

The following covariates are included in the analysis: percentage of the population who were aged 70 or over (using the ONS 2018 mid-year population estimates);[8] population density (number of people per square kilometre, using the ONS 2018 mid-year population estimates);[8] the 2019 Index of Multiple Deprivation score;[10] and levels of pollution as marked by population-weighted annual mean PM2.5 (anthropogenic) concentration for 2018 (ugm-3).[11]

We use linear regression models to predict variation in COVID-19 related death rate across local authorities. First simple, descriptive models are used to show the association between each ethnic minority category and risk of death, and the association between each covariate and risk of death. Then we included all the predictor variables to show the fully-adjusted relationship between COVID-19 related deaths and the proportion of the local population who are ethnic minority.

Patient and Public Involvement

Patients or the public were not involved in the design, or conduct, or reporting, or dissemination plans of this research. The research was based entirely on secondary analysis of existing administrative data.

Results

For peer review only - http://bmjopen.bmj.com/site/about/guidelines.xhtml

Table 1 presents the results of the crude descriptive models, showing unadjusted coefficients for the relationship between proportion of the population in a local authority who are of an ethnic minority group and the COVID-19 related death rate. For ease of interpretation the coefficients have been estimated as the change in number of deaths per million for a 1% rise in proportion of the population who are in the ethnic group. Although descriptive in nature, this analysis indicates a marked inequality between areas, with a rise in the ethnic minority population in an area related to a statistically significant higher rate of COVID-19 related death. This is present for each of the ethnic categories shown in the table, and also present for each sub-category in a disaggregated analysis (not shown but available from the authors). Most confidence can be placed on the estimate for the total ethnic minority population, which combines all of the other groups, because the population size is relatively large and more evenly distributed across local authorities. This coefficient shows that for every 1% rise in proportion of the population who are ethnic minority COVID-19 related deaths increased by 5·12 (4·00 to 6·24) per million.

Table 1: Association between size of ethnic population and COVID-19 deaths at local authority level: England

Death rate per million for 1% increase in population size

Asian	Black	White minority, excluding Irish and Travellers	Other incl· Mixed	Total ethnic minority
8·94<br>(6·67 – 11·21)	19·85<br>(15·33 – 24·38)	10·30<br>(6·03 – 14·58)	33·04<br>(25·51 – 40·57)	5·12<br>(4·00 – 6·24)

Table 2 presents a descriptive analysis of the relationship between covariates and COVID-19 related death rates in a local authority.

Table 2: Association between area characteristics and COVID-19 deaths at local authority level: England

Death rate per million

1% increase in proportion of population aged 70 or older	Population density (100 per square km)	Index of Multiple Deprivation	Pollution
-0·20<br>(-0·26 – -0·15)	0·27<br>(0·19 – 0·35)	0·28<br>(0·084 – 0·56)	0·36<br>(0·25 – 0·47)

Each of these area characteristics are significantly associated with risk of death, with population density, Index of Multiple Deprivation and level of pollution all increasing risk. However, surprisingly, the proportion of the population who are aged 70 or older, a risk factor for COVID-19 related death, is negatively associated with risk of death, although the effect is very small. Subsequent analysis (not shown, but available from the authors) revealed that this was possibility a result of the negative correlation between the proportion of ethnic minority people and the proportion of people aged 70 and over in an area.

Finally, Table 3 presents the full model, with each of the risk factors present. Once all variables are included, only the proportion of the population who are ethnic minority remains statistically

For peer review only - http://bmjopen.bmj.com/site/about/guidelines.xhtml

significant. The estimated coefficient for proportion ethnic minority is a little reduced compared with the unadjusted figure, going from 5·12 (4·00 to 6·24) to 4·42 (2·24 to 6·60), suggesting that some of the ethnic minority association results from ethnic minority people living in more densely populated, more polluted and more deprived areas.

Table 3: Multivariate analysis of association between area characteristics and COVID-19 deaths at local authority level: England

Death rate per million

	1% increase in proportion ethnic minority	1% increase in proportion aged 70 or older	Population density (100 per square km)	Index of Multiple Deprivation	Pollution
Death rate per million	4·42 (2·24 – 6·60)	-0·011	-0·0023	0·098	0·10
Probability	<0·001	0·82	0·73	0·51	0·19

To illustrate the significance of these findings, if we take the average England COVID-19 related death rate as 46·45 per hundred thousand, for a local authority with twice the average number of ethnic minority people (using 2018 ETHPOP estimates this would be an increase from 23% to 46%) would have a predicted COVID-19 related death rate of 58·23 per hundred thousand (46·45+(23*5·12/10)), an increase of 25%. While a local authority with a small proportion of ethnic minority people, say 3% rather than 23%, would have a predicted death rate of 36·21 per hundred thousand (46·45-(20*5·12/10)), a decrease of 22%.

Discussion

The analyses presented here provide the strongest evidence to date on ethnic inequalities in COVID-19 related mortality in England. Findings from these analyses show a clear association between an increase in the proportion of ethnic minority residents in a local authority and an increase in mortality related to COVID-19, a finding that is consistent with other literature.[12] Of importance is that this analysis provides robust and up to date denominators for the ethnic minority population, a comprehensive and recent count of deaths, examines the situation for different categories of ethnic minority groups, and that it includes adjustments for other factors that might explain the association between ethnicity and risk, including those that have been shown to be associated with increased mortality from COVID-19, such as pollution,[13] or that have been found to be risk factors for mortality at the neighbourhood-level, such as deprivation,[12,14] and population density,[12] However, it is important to note that these controls are partial and, because of data limitation, only cover some of the relevant risk factors, which we discuss below.

However, it is important to bare in mind that these analyses are ecological, so inferences cannot be drawn about individuals from these aggregated data. This means that the increased risk of mortality identified may be shared to a certain extent among different ethnic groups in the area, although the evidence of an association between increased risk and each of the categories of ethnic minority group examined is suggestive of the risk not being equally shared between ethnic minority and ethnic majority people living in an area. In addition, we can draw from existing evidence documenting ethnic inequalities across a range of socioeconomic, mental health, and physical health outcomes,[15-17] including non-COVID-19 related mortality,[18] to support the likelihood that ethnic minority residents

For peer review only - http://bmjopen.bmj.com/site/about/guidelines.xhtml

suffer from an increased risk of exposure and vulnerability to COVID-19 infection and related mortality, and whose increased risk, therefore, is likely to be a key factor driving these findings.

There has been much public debate about what might be driving apparent ethnic inequalities in risk of COVID-19 related complications and death. Most of these discussions point towards the role of underlying social and economic inequalities that are faced by ethnic minority people. That is, most ethnic minority groups are more vulnerable to, and have poorer prognosis from, COVID-19 infection, because they are more likely to: be poorer; have poorly paid and insecure employment; live in over-crowded housing; and live in deprived neighbourhoods with high rates of concentrated poverty and increased pollution levels.[19] The fully adjusted model, which shows a reduction in the coefficient related to proportion of the population who are ethnic minority once factors such as these are taken into account, provides some support for these possibilities. However, it is important to note again that these are ecological analyses, so do not account for the very likely possibility that ethnic minority people have poorer circumstances than others living in the same area.

Ethnic minority people are also more likely to be employed in sectors that increase their risk of exposure to COVID-19. An over-representation of ethnic minority people can be found working in transport and delivery jobs, as health care assistants, hospital cleaners, social care workers, and in nursing and medical jobs.[20] Not only do these occupations increase risk of infection,[20] some of these are also occupations that have been the last to receive supplies of personal protective equipment. People in these occupations have now been deemed key workers, but for decades ethnic minority people working in these jobs have endured job insecurity, low pay, and discrimination.

In addition, ethnic minority people are more likely to have the underlying health conditions that have been linked to increased risk of COVID-19 related complications and mortality, such as asthma, diabetes, high blood pressure, and coronary heart disease.[21] These health conditions are socially-patterned, so that the social and economic inequalities faced by ethnic minority people described above, lead to an increased risk of developing these health conditions.[15] As a result, we would argue that the increased risks associated with COVID-19 infection faced by ethnic minority people are now a core component of wider ethnic inequalities in health, and these negative consequences are amplified by long established pre-existing ethnic inequalities in health, both of which are driven by social and economic inequalities.

Behind this complexity, however, is a key consideration that is typically absent from investigations into ethnic inequalities in health – that the social and economic inequalities that are faced by ethnic minority people are driven by entrenched structural and institutional racism and racial discrimination.[22] An explanation of ethnic inequalities that stops at social and economic inequalities and doesn't acknowledge how these inequalities have been, and continue to be, shaped by historical processes underpinned by racism, will be limited in its ability to generate an understanding of, and solutions to, ethnic inequalities. A myriad of studies in the UK and elsewhere have now documented the role of racism in patterning inequalities in education, employment, income, housing, and proximity to pollution.[23-27] In addition, experiences of racial discrimination have been linked to a numerous mental and physical health outcomes, including asthma and hypertension.[17,22,28] Importantly, these processes do not operate in isolation, they co-occur and sequentially lead to deepening inequalities in many domains across a person's life course, and are transmitted from one generation to the next.[29,30]

Excluding racism – the root of ethnic inequalities in COVID-19 infections and related mortality – from scientific and policy discussions around the determinants and implications of the coronavirus pandemic can lead to dangerous and ineffective investigations and policy interventions. These include un-evidenced reductionist approaches which question whether ethnic inequalities in COVID-19 may

For peer review only - http://bmjopen.bmj.com/site/about/guidelines.xhtml

be due to biological/genetic or cultural differences, a line of thinking that risks taking us back into a time of scientific racism, but which is, for example, reflected in a recent call for research on this issue.[31] Before we respond to such an agenda we should ask ourselves the simple question: 'what could possibly be the biological or cultural similarities between an ethnic minority family living in Tower Hamlets, London and another living in Detroit, Michigan, both of whom face an increased risk of COVID-19 related complications and mortality?' More likely than shared genetic and cultural risks, is that they will both live in disinvested neighbourhoods with high levels of pollution and concentrated poverty, with insecure and underpaid employment, and in overcrowded conditions with substandard levels of housing. Chances are they have had their lives shaped by institutional and structural racism, and have experiences of racial discrimination deeply embedded in their lives.[15-19,22] These are the similarities that policy and research efforts should be paying attention to. Given this, the increased risks faced by ethnic minority people should not have been unexpected, as appears to have been the case, they could and should have been anticipated.

That Public Health England is reviewing ethnic inequalities in COVID-19 related outcomes could be an important shift of focus, especially when contemporary policy work around inequalities in health have largely ignored the question of ethnicity.[32,33] However, it is crucial that this review considers how current inequalities relate to longstanding ethnic inequalities in health and in doing so the question of racism must not be side-stepped. Similarly, the review must also focus on the greater harm done to ethnic minority people as a result of Government responses to the coronavirus pandemic and move quickly to consider how these greater harms might be mitigated. The justification for the pandemic measures is that their estimated effect on reducing the impact of the COVID-19 pandemic on the NHS, by protecting its capacity to provide care for people who become seriously ill as a result of a COVID-19 infection, would offset their acknowledged extremely negative economic, social, health and psychological impacts. That is, the negative is on average judged to be worth the estimated direct health benefits. However, the situation facing ethnic minority people is far more precarious than 'the average', as detailed above, meaning that these measures are certainly having a more negative effect on ethnic minority people. In addition, some of the more punitive dimensions of 'lockdown', such as changes in the Mental Health Act,[34] police surveillance, and discontinuity in the clinical management of pre-existing conditions, are also going to more adversely impact those with racialised identities.

References

1. ICNARC report on COVID-19 in critical care 24 April 2020, https://www.icnarc.org/Our-Audit/Audits/Cmp/Reports, accessed 26th April
2. Ethnic minorities dying of Covid-19 at higher rate, analysis shows. The Guardian, 22 April 2020. Available at: https://www.theguardian.com/world/2020/apr/22/racial-inequality-in-britain-found-a-risk-factor-for-covid-19
3. Cook T, Kursumovic E, Lennane S. Exclusive: deaths of NHS staff from covid-19 analysed. Health Serv J 2020 Apr 22. https://www.hsj.co.uk/exclusive-deaths-of-nhs-staff-fromcovid-19-analysed/7027471.article
4. Platt, L. and Warwick R. (2020) Are some ethnic groups more vulnerable to COVID-19 than others? Institute for Fiscal Studies ISBN 978-1-912805-75-4
5. Thebault R, Tran AB, Williams V. The coronavirus is infecting and killing black Americans at an alarmingly high rate. Washington Post 2020 Apr 7. https://www.washingtonpost.com/nation/2020/04/07/coronavirus-is-infecting-killing-black-americans-an-alarmingly-high-rate-post-analysis-shows
6. https://www.propublica.org/article/early-data-shows-african-americans-have-contracted-and-died-of-coronavirus-at-an-alarming-rate
7. https://www.ons.gov.uk/peoplepopulationandcommunity/healthandsocialcare/causesofdeath/datasets/deathregistrationsandoccurrencesbylocalauthorityandhealthboard, accessed 28th April

For peer review only - http://bmjopen.bmj.com/site/about/guidelines.xhtml

8. https://www.ons.gov.uk/peoplepopulationandcommunity/populationandmigration/populationestimates/datasets/populationestimatesforukenglandandwalesscotlandandnorthernireland
9. Wohland P, Burkitt M, Norman P, Rees P, Boden P and Durham H, ETHPOP Database, ESRC Follow on Fund "Ethnic group population trends". www.ethpop.org. Date of extraction 26th April 2020.
10. https://assets.publishing.service.gov.uk/government/uploads/system/uploads/attachment_data/file/835115/IoD2019_Statistical_Release.pdf
11. https://uk-air.defra.gov.uk/data/pcm-data#population_weighted_annual_mean_pm25_data
12. Bray I., Gibson A., White, J. Coronavirus disease 2019 mortality: a multivariate ecological analysis in relation to ethnicity, population density, obesity, deprivation and pollution. Public Health, 2020, 185, 261-263
13. Wu, X., Nethery, R., Sabath, B., Braun, D., & Dominici, F. (2020). Exposure to air pollution and COVID-19 mortality in the United States medRxiv 2020.04.05.20054502; doi: https://doi.org/10.1101/2020.04.05.20054502
14. ICNARC. ICNARC report on COVID-19 in critical care. ICNARC COVID-19 Study Case Mix Program Database 2020;:1–16.https://www.icnarc.org/Our-Audit/Audits/Cmp/Reports.
15. Nazroo J. Y. (2003). The structuring of ethnic inequalities in health: economic position, racial discrimination, and racism. American journal of public health, 93(2), 277–284. https://doi.org/10.2105/ajph.93.2.277
16. Bécares, L. (2015). Which ethnic groups have the poorest health?. In Jivraj, S. and Simpson, L. (eds.) Ethnic identity and inequalities in Britain. Bristol: The Policy Press
17. Wallace, S., Nazroo, J.Y. and Bécares, L. (2016)Cumulative exposure to racial discrimination across time and domains: exploring racism's long term impact on the mental health of ethnic minority people in the UK. American Journal of Public Health, 106(7), 1294-1300. doi: 10.2105/AJPH.2016.303121
18. Bos, V., Kunst, A., Keij-Deerenberg, I., Garssen, J., & Mackenbach, J. (2004). Ethnic inequalities in age- and cause-specific mortality in The Netherlands, International Journal of Epidemiology, 33(5), 1112–1119.
19. Byrne, B., Alexander, C., Khan, O., Nazroo, J. And Shankley, W. (eds.) (2020) Ethnicity, Race and Inequality in the UK: State of the Nation, Bristol: Policy Press ISBN 978-1447351269)
20. Office for National Statistics. Coronavirus (COVID-19) related deaths by occupation, England and Wales deaths registered between 9 March and 25 May 2020. 2020. https://www.ons.gov.uk/peoplepopulationandcommunity/healthandsocialcare/causesofdeath/bulletins/coronaviruscovid19relateddeathsbyoccupationenglandandwales/deathsregisteredbetween9marchand25may2020
21. Nazroo, J.Y. (1997) The health of Britain's ethnic minorities: findings from a national survey, London: Policy Studies Institute ISBN 0 85374 709 1
22. Nazroo, J., Bhui, K. and Rhodes, J. (2020) 'Where next for understanding race/ethnic inequalities in severe mental illness? Structural, interpersonal and institutional racism', *Sociology of Health and Illness*, 42, 2, 262-276, doi: 10.1111/1467-9566.130012
23. Alexander, C. and Arday, J. (eds) (2015) Aiming Higher: Race Inequality and Diversity in the Academy.London: Runnymede Trust.
24. Zschirnt, E. and Ruedin, D. (2016): Ethnic discrimination in hiring decisions: a meta-analysis of correspondence tests 1990–2015. Journal of Ethnic and Migration Studies 42(7): 1115-34.
25. Ashe, S. and Nazroo, J. (2016) Equality, Diversity and Racism in the Workplace: A Qualitative Analysis of the 2015 Race at Work Survey, London: Business in The Community, https://race.bitc.org.uk/system/files/research/race_at_work_equality_diversity_and_racism_in_the_workplace_november_2016.pdf
26. Hogan, Bernie, and Brent Berry. 2011. "Racial and Ethnic Biases in Rental Housing: An Audit Study of Online Apartment Listings." City and Community 10 (4): 351–372.

For peer review only - http://bmjopen.bmj.com/site/about/guidelines.xhtml

27. Cohen, H. W., & Northridge, M. E. (2008). Getting political: racism and urban health. American journal of public health, 98(9 Suppl), S17–S19. https://doi.org/10.2105/ajph.98.supplement_1.s17
28. Bécares, L., Stafford, M., & Nazroo, J. (2009). Fear of racism, employment and expected organizational racism: their association with health. European Journal of Public Health, 19(5), 504-510.
29. Gee, G. C., Walsemann, K. M., & Brondolo, E. (2012). A life course perspective on how racism may be related to health inequities. American journal of public health, 102(5), 967–974. https://doi.org/10.2105/AJPH.2012.300666
30. Bécares, L., Nazroo, J., & Kelly, Y. (2015). A longitudinal examination of maternal, family, and area-level experiences of racism on children's socioemotional development: patterns and possible explanations. Social Science & Medicine, 142, 128-135
31. NIHR and UKRI Launch call for research on COVID-19 and ethnicity. Available at: https://www.nihr.ac.uk/news/nihr-and-ukri-launch-call-for-research-on-covid-19-and-ethnicity/24658
32. https://www.bmj.com/content/368/bmj.m268.full%22
33. Salway, S., Holman, D., Lee, C., McGowan, V., Ben-Shlomo, Y., Saxena, S. and Nazroo, J. (2020) 'Transforming the health system for the UK's multi-ethnic population', British Medical Journal, 368, m268 doi: 10.1136/bmj.m268
34. https://synergicollaborativecentre.co.uk/new-coronavirus-act-may-increase-the-mental-health-detention-of-ethnic-minority-people/%22

Acknowledgements

We are grateful for the support provided by Caelainn Barr, Editor, Data projects, Guardian News and Media, and Niamh McIntyre, Niko Kommenda and Antonio Voce, Data projects, Guardian News and Media.

Funding

Nazroo's work on this paper was funded by the Economic and Social Research Council, grant number ES/R009341/1.

Data sharing

The analyses presented here make use of publicly available data. Links to the data are provided within the paper.

For peer review only

For peer review only - http://bmjopen.bmj.com/site/about/guidelines.xhtml

STROBE Statement—Checklist of items that should be included in reports of ***cross-sectional studies***

	Item No	Recommendation	Page No
**Title and abstract**	1	(*a*) Indicate the study's design with a commonly used term in the title or the abstract	1
		(*b*) Provide in the abstract an informative and balanced summary of what was done and what was found	2
**Introduction**			
Background/rationale	2	Explain the scientific background and rationale for the investigation being reported	3
Objectives	3	State specific objectives, including any prespecified hypotheses	3
**Methods**			
Study design	4	Present key elements of study design early in the paper	3-4
Setting	5	Describe the setting, locations, and relevant dates, including periods of recruitment, exposure, follow-up, and data collection	3-4
Participants	6	(*a*) Give the eligibility criteria, and the sources and methods of selection of participants	N/a
Variables	7	Clearly define all outcomes, exposures, predictors, potential confounders, and effect modifiers. Give diagnostic criteria, if applicable	4
Data sources/ measurement	8*	For each variable of interest, give sources of data and details of methods of assessment (measurement). Describe comparability of assessment methods if there is more than one group	4
Bias	9	Describe any efforts to address potential sources of bias	N/a
Study size	10	Explain how the study size was arrived at	N/a
Quantitative variables	11	Explain how quantitative variables were handled in the analyses. If applicable, describe which groupings were chosen and why	4
Statistical methods	12	(*a*) Describe all statistical methods, including those used to control for confounding	4
		(*b*) Describe any methods used to examine subgroups and interactions	4
		(*c*) Explain how missing data were addressed	N/a
		(*d*) If applicable, describe analytical methods taking account of sampling strategy	4
		(*e*) Describe any sensitivity analyses	N/a
**Results**			
Participants	13*	(a) Report numbers of individuals at each stage of study—eg numbers potentially eligible, examined for eligibility, confirmed eligible, included in the study, completing follow-up, and analysed	N/a
		(b) Give reasons for non-participation at each stage	N/a
		(c) Consider use of a flow diagram	N/a
Descriptive data	14*	(a) Give characteristics of study participants (eg demographic, clinical, social) and information on exposures and potential confounders	N/a
		(b) Indicate number of participants with missing data for each variable of interest	N/a
Outcome data	15*	Report numbers of outcome events or summary measures	4
Main results	16	(*a*) Give unadjusted estimates and, if applicable, confounder-adjusted estimates and their precision (eg, 95% confidence interval). Make clear which confounders were adjusted for and why they were included	5-6

For peer review only

		(*b*) Report category boundaries when continuous variables were categorized	N/a
		(*c*) If relevant, consider translating estimates of relative risk into absolute risk for a meaningful time period	N/a
Other analyses	17	Report other analyses done—eg analyses of subgroups and interactions, and sensitivity analyses	5-6
**Discussion**			
Key results	18	Summarise key results with reference to study objectives	6
Limitations	19	Discuss limitations of the study, taking into account sources of potential bias or imprecision. Discuss both direction and magnitude of any potential bias	6-7
Interpretation	20	Give a cautious overall interpretation of results considering objectives, limitations, multiplicity of analyses, results from similar studies, and other relevant evidence	6-8
Generalisability	21	Discuss the generalisability (external validity) of the study results	N/a
**Other information**			
Funding	22	Give the source of funding and the role of the funders for the present study and, if applicable, for the original study on which the present article is based	1

*Give information separately for exposed and unexposed groups.

Note: An Explanation and Elaboration article discusses each checklist item and gives methodological background and published examples of transparent reporting. The STROBE checklist is best used in conjunction with this article (freely available on the Web sites of PLoS Medicine at http://www.plosmedicine.org/, Annals of Internal Medicine at http://www.annals.org/, and Epidemiology at http://www.epidem.com/). Information on the STROBE Initiative is available at www.strobe-statement.org.

For peer review only

For peer review only - http://bmjopen.bmj.com/site/about/guidelines.xhtml

BMJ Open

Evidence for ethnic inequalities in mortality related to COVID-19 infections: Findings from an ecological analysis of England

Journal:	*BMJ Open*
Manuscript ID	bmjopen-2020-041750.R2
Article Type:	Original research
Date Submitted by the Author:	21-Oct-2020
Complete List of Authors:	Nazroo, James; The University of Manchester, Sociology<br>Becares, Laia; University of Sussex, Social Work and Social Care
**Primary Subject Heading**:	Epidemiology
Secondary Subject Heading:	Public health, Sociology
Keywords:	EPIDEMIOLOGY, Epidemiology < INFECTIOUS DISEASES, Public health < INFECTIOUS DISEASES, PUBLIC HEALTH

SCHOLARONE™
Manuscripts

I, the Submitting Author has the right to grant and does grant on behalf of all authors of the Work (as defined in the below author licence), an exclusive licence and/or a non-exclusive licence for contributions from authors who are: i) UK Crown employees; ii) where BMJ has agreed a CC-BY licence shall apply, and/or iii) in accordance with the terms applicable for US Federal Government officers or employees acting as part of their official duties; on a worldwide, perpetual, irrevocable, royalty-free basis to BMJ Publishing Group Ltd ("BMJ") its licensees and where the relevant Journal is co-owned by BMJ to the co-owners of the Journal, to publish the Work in this journal and any other BMJ products and to exploit all rights, as set out in our licence.

The Submitting Author accepts and understands that any supply made under these terms is made by BMJ to the Submitting Author unless you are acting as an employee on behalf of your employer or a postgraduate student of an affiliated institution which is paying any applicable article publishing charge ("APC") for Open Access articles. Where the Submitting Author wishes to make the Work available on an Open Access basis (and intends to pay the relevant APC), the terms of reuse of such Open Access shall be governed by a Creative Commons licence – details of these licences and which Creative Commons licence will apply to this Work are set out in our licence referred to above.

Other than as permitted in any relevant BMJ Author's Self Archiving Policies, I confirm this Work has not been accepted for publication elsewhere, is not being considered for publication elsewhere and does not duplicate material already published. I confirm all authors consent to publication of this Work and authorise the granting of this licence.

For peer review only

Evidence for ethnic inequalities in mortality related to COVID-19 infections: Findings from an ecological analysis of England

James Nazroo (corresponding author)
Professor of Sociology
Centre on Dynamics of Ethnicity
University of Manchester
Humanities Building
Oxford Road
Manchester M13 9PL
Email: james.nazroo@manchester.ac.uk

Laia Bécares
Senior Lecturer in Applied Social Sciences
Social Work and Social Care
University of Sussex
Essex House, Falmer, Brighton, BN1 9QQ
Email: L.Becares@sussex.ac.uk

Contributorship statement

J. Nazroo and L. Bécares were both involved in the design of the study, interpretation of the findings, and writing of the manuscript. J. Nazroo carried out the analysis. The final manuscript was approved by both J. Nazroo and L. Bécares.

Competing interests

There are no competing interests for any author.

For peer review only - http://bmjopen.bmj.com/site/about/guidelines.xhtml

Abstract

Objectives
In the absence of robust direct data on ethnic inequalities in COVID-19 related mortality in the UK, we examine the relationship between ethnic composition of an area and rate of mortality in the area.

Design
Ecological analysis of COVID-19 related mortality rates occurring by 24th April 2020, and ethnic composition of the population. Account is taken of age, population density, area deprivation and pollution.

Setting
Local authorities in England.

Results
For every 1% rise in proportion of the population who are ethnic minority, COVID-19 related deaths increased by 5·12, 95% CI [4·00, 6·24], per million. This rise is present for each ethnic minority category examined, including the White minority group. The size of this increase is a little reduced in an adjusted model to 4·42, 95% CI [2·24, 6·60], suggesting that some of the association results from ethnic minority people living in more densely populated, more polluted and more deprived areas.

This estimate suggests that the average England COVID-19 related death rate would rise by 25% in a local authority with twice the average number of ethnic minority people.

Conclusions
We find clear evidence that rates of COVID-19 related mortality within a local authority increases as the proportion of the population who are ethnic minority increases. We suggest that this is a consequence of social and economic inequalities driven by entrenched structural and institutional racism and racial discrimination. We argue that these factors should be central to any investigation of ethnic inequalities in COVID-19 outcomes.

Strengths and Limitations

- The analysis includes a comprehensive and recent count of deaths, including those occurring outside of clinical settings.
- The situation for different categories of ethnic minority group, including a white minority group, is examined.
- Robust and up to date denominators for the ethnic minority population are used, rather than relying on out of date or inaccurate estimates.
- Partial adjustments have been made for other factors that might explain the association between ethnicity and risk, including those that have been shown to be associated with increased mortality from COVID-19, such as pollution, or that have been found to be risk factors for mortality at the neighbourhood-level, such as deprivation and population density.
- However, these analyses are ecological, so inferences cannot be drawn about individuals from these aggregated data and the increased risk of mortality identified may be shared to a certain extent among different ethnic groups in an area.

For peer review only - http://bmjopen.bmj.com/site/about/guidelines.xhtml

Introduction

There is a growing body of evidence suggesting that there are marked ethnic inequalities in COVID-19 related deaths. Although UK data on ethnicity in relation to COVID-19 are sparse (as are data on ethnicity and health more generally), a report by The Intensive Care National Audit and Research Centre showed that around 35% of COVID-19 related admissions to intensive care were ethnic minority people and ethnic minority admissions were slightly more like to die in critical care (for example, 48·4% of White patients died in critical care compared with 55·3% of ethnic minority patients).[1] Further evidence published by the Guardian and Times newspapers, based on their own, non-peer reviewed research, suggested that ethnic minority people represent 19% of deaths recorded in hospital,[2] and that areas with a higher proportion of non-white ethnic minority people had higher death rates,[2] while, recent analysis of 106 healthcare workers who have died from covid-19 showed that 63% were from an ethnic minority background, and just over half were not born in the UK.[3] Given that non-white ethnic minority people made up 14% of the population in England and Wales at the 2011 Census, this suggests a marked inequality. This impression was reinforced by secondary analysis of data released by the NHS, which suggested meaningful increases in death rates for ethnic minority people after taking into account differences in age structures and place of residence.[4] Evidence of ethnic/race inequalities in relation to COVID-19 has also been reported in the United States, where regional analyses indicate that areas with larger ethnic minority populations have higher rates of both COVID-19 infections and related deaths.[5] For example, in Michigan, a state where 15% of the population is Black, 40% of deaths are of Black people.[6]

Despite this accumulating evidence, it is hard to draw firm conclusions on the extent of ethnic inequalities in COVID-19 related risks. A large proportion of the evidence discussed in media reports is impressionistic, and where statistics on admissions or deaths are collated, denominators are often missing, or are crudely estimated at a national level, rather than estimated from the population from which deaths are counted. In addition, the national data used to calculate denominators are typically drawn from the 2011 Census, which is considerably out of date and almost certainly underestimates the size of the ethnic minority population. Importantly, adjustments are rarely made for the younger age profile of ethnic minority people, nor for the potentially increased exposure to COVID-19 infection that results from their greater concentration in areas with a high population density, or greater risk from infection associated with higher levels of pollution and living in areas with greater levels of deprivation. In addition, the data published rarely differentiate between different ethnic groups, and largely ignore White minority groups, meaning that potential differences between different ethnic minority groups are missed. Finally, the count of deaths often only includes those occurring in a hospitals, disregarding those who died in community settings.

To begin to address these issues and to enhance our understanding of ethnic inequalities in COVID-19 related risks, we conduct an ecological analysis, at local authority level, of the relationship between COVID-19 related deaths and the proportion of the local population who are ethnic minority. To do this we use the most recent release of data at the time of writing, accurate estimates of the size and ethnic composition of the population in local authorities, and a modelling approach that accounts for some potential explanations, noted above, for the higher risk faced by ethnic minority people. We note that the approach to explanation is partial, with both incomplete coverage of potential explanatory factors and risk of ecological fallacy.

Methods

Death rates are taken from ONS 5th May 2020 release of deaths in England, by local authority.[7] (Local authorities are government organisations officially responsible for all the public services and facilities

For peer review only - http://bmjopen.bmj.com/site/about/guidelines.xhtml

for both individuals and business in an area.) This release includes all deaths, including those that occurred in community settings, that occurred up to 24th April and that were registered by the 2nd May. Only those deaths recorded as involving COVID-19, a recording that is based on any mention of COVID-19 on the death certificate, are included. These figures do not include deaths of those who reside outside England, or deaths where the place of residence is either missing or not yet fully coded on the death certificate. There were 26,004 COVID-19 related deaths in England by 24th April, as recorded on 2nd May.

To provide the denominator to calculate COVID-19 related death rates by local authority the ONS 2018 mid-year population estimates are used.[8] These data estimate the England population as 55,977,178, meaning that the COVID-19 related death rate for England was 46·45 per 100,000 as of 24th April 2020.

To examine the association between COVID-19 related deaths and proportion of the local population who are ethnic minority across local authorities, we use linear regression models with COVID-19 related death rate as the dependent variable. The main predictor variable, the proportion of the population who are ethnic minority, was taken from the ETHPOP estimates for 2018 (we used the 2018 used rather than those for 2020 in order to match in time with the ONS population estimates used for total population size).[9] These estimates are based on sophisticated modelling of change in the ethnic composition of the population at local authority level, accounting for births, deaths and migration both into the UK and within the UK. This is done separately for different ethnic groups, the categories used being: White British/Irish/Traveller; Indian; Pakistani; Bangladeshi; Chinese; Other Asian; Black African; Black Caribbean; Other Black; Mixed; White Other; and Other. For 2018 this model estimates that the England population was 77.2% White British/Irish/Traveller. Given the relatively small population sizes of ethnic minority groups and their concentration in particular regions of England, the analysis conducted here combine ethnic minority groups into four categories: Asian (Indian, Pakistani, Bangladeshi, Chinese, Other Asian); Black (Black African, Black Caribbean, Other Black); White Other; and Other (Mixed, Other). For the more detailed modelling all of the ethnic minority groups are combined together and compared with the White British/Irish/Traveller group. In each case, these variables are modelled as a continuous variable that captures a 1% increase in the concentration of the group in a local authority.

The following covariates are included in the analysis: percentage of the population who were aged 70 or over (using the ONS 2018 mid-year population estimates);[8] population density (number of people per square kilometre, using the ONS 2018 mid-year population estimates);[8] the 2019 Index of Multiple Deprivation score;[10] and levels of pollution as marked by population-weighted annual mean PM2.5 (anthropogenic) concentration for 2018 (ugm-3).[11]

We use linear regression models to predict variation in COVID-19 related death rate across local authorities. First simple, descriptive models are used to show the association between each ethnic minority category and risk of death, and the association between each covariate and risk of death. Then we included all the predictor variables to show the fully-adjusted relationship between COVID-19 related deaths and the proportion of the local population who are ethnic minority.

Patient and Public Involvement

Patients or the public were not involved in the design, conduct, reporting, or dissemination plans of this research. The research was based entirely on secondary analysis of existing administrative data.

For peer review only - http://bmjopen.bmj.com/site/about/guidelines.xhtml

Results

Table 1 presents the results of the crude descriptive models, showing unadjusted coefficients for the relationship between proportion of the population in a local authority who are of an ethnic minority group and the COVID-19 related death rate. For ease of interpretation the coefficients have been estimated as the change in number of deaths per million for a 1% rise in proportion of the population who are in the ethnic group. Although descriptive in nature, this analysis indicates a marked inequality between areas, with a rise in the ethnic minority population in an area related to a statistically significant higher rate of COVID-19 related death. This is present for each of the ethnic categories shown in the table, and also present for each sub-category in a disaggregated analysis (not shown but available from the authors). Most confidence can be placed on the estimate for the total ethnic minority population, which combines all of the other groups, because the population size is relatively large and more evenly distributed across local authorities. This coefficient shows that for every 1% rise in proportion of the population who are ethnic minority COVID-19 related deaths increased by 5·12, 95% CI [4·00, 6·24], per million.

Table 1: Association between size of ethnic population and COVID-19 deaths at local authority level: England

	Asian	Black	White minority, excluding Irish and Travellers	Other incl· Mixed	Total ethnic minority
Death rate per million	8·94*	19·85*	10·30*	33·04*	5·12*
95% confidence interval	[6·67, 11·21]	[15·33, 24·38]	[6·03, 14·58]	[25·51, 40·57]	[4·00, 6·24]

*p<0.05

Table 2 presents a descriptive analysis of the relationship between covariates and COVID-19 related death rates in a local authority.

Table 2: Association between area characteristics and COVID-19 deaths at local authority level: England

	1% increase in proportion aged 70 or older	Population density (100 per square km)	Index of Multiple Deprivation	Pollution
Death rate per million	-0·20*	0·27*	0·28*	0·36*
95% confidence interval	[-0·26, -0·15]	[0·19, 0·35]	[0·084, 0·56]	[0·25, 0·47]

*p<0.05

Each of these area characteristics are significantly associated with risk of death, with population density, Index of Multiple Deprivation and level of pollution all increasing risk. However, surprisingly, the proportion of the population who are aged 70 or older, a risk factor for COVID-19 related death,

For peer review only - http://bmjopen.bmj.com/site/about/guidelines.xhtml

is negatively associated with risk of death, although the effect is very small. Subsequent analysis (not shown, but available from the authors) revealed that this was possibility a result of the negative correlation between the proportion of ethnic minority people and the proportion of people aged 70 and over in an area.

Finally, Table 3 presents the full model, with each of the risk factors present. Once all variables are included, only the proportion of the population who are ethnic minority remains statistically significant. The estimated coefficient for proportion ethnic minority is a little reduced compared with the unadjusted figure, going from 5·12, 95% CI [4·00, 6·24], to 4·42, 95% CI [2·24, 6·60], suggesting that some of the ethnic minority association results from ethnic minority people living in more densely populated, more polluted and more deprived areas.

Table 3: Multivariate analysis of association between area characteristics and COVID-19 deaths at local authority level: England

	1% increase in proportion ethnic minority	1% increase in proportion aged 70 or older	Population density (100 per square km)	Index of Multiple Deprivation	Pollution
Death rate per million	4·42*	-0·011	-0·0023	0·098	0·10
95% confidence interval	[2·24, 6·60]	[-0.11, 0.083]	[-0.015, 0.011]	[-0.194, 0.389]	[-0.05, 0.251]

*p<0.05

To illustrate the significance of these findings, if we take the average England COVID-19 related death rate as 46·45 per hundred thousand, for a local authority with twice the average number of ethnic minority people (using 2018 ETHPOP estimates this would be an increase from 23% to 46%) would have a predicted COVID-19 related death rate of 58·23 per hundred thousand (46·45+(23*5·12/10)), an increase of 25%. While a local authority with a small proportion of ethnic minority people, say 3% rather than 23%, would have a predicted death rate of 36·21 per hundred thousand (46·45-(20*5·12/10)), a decrease of 22%.

Discussion

Key findings

The analyses presented here provide the strongest evidence to date on ethnic inequalities in COVID-19 related mortality in England. Findings from these analyses show a clear association between an increase in the proportion of ethnic minority residents in a local authority and an increase in mortality related to COVID-19, a finding that is consistent with other literature.[12] The increased risk diminishes slightly, but remains, after controlling for some other characteristics of the area: age profile, population density, level of deprivation and pollution.

Strengths and limitations

Strengths of this analysis are that it provides robust and up to date denominators for the ethnic minority population, a comprehensive and recent count of deaths, examines the situation for different categories of ethnic minority groups, and that it includes adjustments for other factors that might explain the association between ethnicity and risk, including those that have been shown to be

For peer review only - http://bmjopen.bmj.com/site/about/guidelines.xhtml

associated with increased mortality from COVID-19, such as pollution,[13] or that have been found to be risk factors for mortality at the neighbourhood-level, such as deprivation,[12,14] and population density,[12] However, it is important to note that these controls are partial and, because of data limitation, only cover some of the relevant risk factors, which we discuss below. Other unmeasured factors may well be relevant to risk of COVID-19 related mortality and may contribute to ethnic inequalities in this risk.

Other limitations to be considered include that these analyses are ecological, so inferences cannot be drawn about individuals from these aggregated data. This means that the increased risk of mortality identified may be shared to a certain extent among different ethnic groups in the area, although the evidence of an association between increased risk and each of the categories of ethnic minority group examined is suggestive of the risk not being equally shared between ethnic minority and ethnic majority people living in an area. In addition, we can draw from existing evidence documenting ethnic inequalities across a range of socioeconomic, mental health, and physical health outcomes,[15-17] including non-COVID-19 related mortality,[18] to support the likelihood that ethnic minority residents suffer from an increased risk of exposure and vulnerability to COVID-19 infection and related mortality, and whose increased risk, therefore, is likely to be a key factor driving these findings.

Interpretation

There has been much public debate about what might be driving apparent ethnic inequalities in risk of COVID-19 related complications and death. As we have previously described,[19] one existing focus has been on the role of the underlying social and economic inequalities that are experienced by ethnic minority people. These inequalities mean that on average ethnic minority people are more likely to: be poorer; have poorly paid and insecure employment; live in over-crowded housing; and live in deprived neighbourhoods with high rates of concentrated poverty and increased pollution levels.[20] This, then, results in greater vulnerability to and poorer prognosis from COVID-19 infection. The adjusted model, which shows a reduction in the coefficient related to proportion of the population who are ethnic minority once factors such as these are taken into account, provides some support for these possibilities. However, it is important to note again that these are ecological analyses, so do not account for the very likely possibility that ethnic minority people have poorer circumstances than others living in the same area.

We have also previously described how ethnic minority people are also more likely to be employed in sectors that increase their risk of exposure to COVID-19,[19] such as in transport and delivery jobs, or working as health care assistants, hospital cleaners, social care workers, and in nursing and medical jobs.[21] Not only do these occupations increase risk of infection,[21] some of these are also occupations that have been the last to receive supplies of personal protective equipment. People in these occupations are now considered to be key workers, but for decades ethnic minority people working in these jobs have endured job insecurity, low pay, and discrimination.

In addition, as noted by ourselves and others,[19] ethnic minority people are more likely to have chronic health conditions that have been linked to increased risk of COVID-19 related complications and mortality, such as asthma, diabetes, high blood pressure, and coronary heart disease.[22] It is important to note that these health conditions are socially-patterned, so the social and economic inequalities faced by ethnic minority people described above lead to their increased prevalence of these health conditions.[15] As a result, we repeat our previously made suggestion that the increased risks associated with COVID-19 infection faced by ethnic minority people are now a core component of wider ethnic inequalities in health, and these negative consequences are amplified by long established pre-existing ethnic inequalities in health, both of which are driven by social and economic inequalities.[19]

In recognising this complexity it is also vital to focus on how the social and economic inequalities that are faced by ethnic minority people are driven by entrenched structural and institutional racism and

For peer review only - http://bmjopen.bmj.com/site/about/guidelines.xhtml

racial discrimination.[23] As we have argued,[19] even though discussion of racism is typically absent from investigations into ethnic inequalities in health, an explanation of ethnic inequalities that stops at social and economic inequalities and doesn't acknowledge how these inequalities have been, and continue to be, shaped by historical processes underpinned by racism, will be limited in its ability to generate an understanding of, and solutions to, ethnic inequalities. A significant and broad body of research has now documented the role of racism in patterning inequalities in education, employment, income, housing, and proximity to pollution.[24-28] In addition, a large number of mental and physical health outcomes, including asthma and hypertension, have been shown to be associated with experiences of racism and discrimination.[17,23,29] Importantly, experiences of racism and discrimination are not one-off, they co-occur and sequentially lead to deepening inequalities in many domains across a person's life course, and are transmitted from one generation to the next.[30,31]

Policy implications and directions for future research

We have previously discussed how excluding racism – the root of ethnic inequalities in COVID-19 infections and related mortality – from scientific and policy discussions around the determinants and implications of the coronavirus pandemic can lead to ineffective investigations and policy interventions.[19] These include un-evidenced approaches that question whether ethnic inequalities in COVID-19 may be due to genetic or cultural differences, a line of thinking that risks taking us back into a time of scientific racism, but which is, for example, reflected in a call for research on this issue.[32] We have suggested that before responding to this agenda we should ask ourselves the simple question: 'What could possibly be the genetic or cultural similarities between an ethnic minority family living in Tower Hamlets, London and another living in Detroit, Michigan, both of whom face an increased risk of COVID-19 related complications and mortality?'.[19] More likely than shared genetic and cultural risks, is that they will both have an increased risk of living in disinvested neighbourhoods with high levels of pollution and concentrated poverty, with insecure and underpaid employment, and in overcrowded conditions with substandard levels of housing. Chances are they have had their lives shaped by institutional and structural racism, and have experiences of racial discrimination deeply embedded in their lives.[15-18,20,23] These are the similarities that policy and research efforts should be paying attention to. Given this, the increased risks faced by ethnic minority people should not have been unexpected, as appears to have been the case, they could and should have been anticipated.

That Public Health England carried out a review of ethnic inequalities in COVID-19 related outcomes could have been an important shift of focus, especially when contemporary policy work around inequalities in health have largely ignored the question of ethnicity.[33,34] However, for that shift to take place it is crucial that ongoing policy work informing Government responses to the coronavirus pandemic considers how current inequalities relate to longstanding ethnic inequalities in health and does not side-step the question of racism, as one Government advisor appear to be attempting to do.[35] Similarly, policy development must also focus on the greater harm done to ethnic minority people as a result of Government responses to the coronavirus pandemic and, as the cycle of responses develops, we should move quickly to consider how these greater harms might be mitigated. The justification for the pandemic measures is that their estimated effect on reducing the impact of the COVID-19 pandemic on the NHS, by protecting its capacity to provide care for people who become seriously ill as a result of a COVID-19 infection, would offset their acknowledged extremely negative economic, social, health and psychological impacts. That is, the negative is on average judged to be worth the estimated direct health benefits. However, as we have described elsewhere,[19] the situation facing ethnic minority people is far more precarious than 'the average', meaning that these measures are certainly having a more negative effect on ethnic minority people. In addition, some of the more punitive dimensions of 'lockdown', such as changes in the Mental Health Act,[36] police surveillance, and discontinuity in the clinical management of pre-existing conditions, are also going to more adversely impact those with racialised identities.

For peer review only - http://bmjopen.bmj.com/site/about/guidelines.xhtml

References

1. ICNARC report on COVID-19 in critical care 24 April 2020, https://www.icnarc.org/Our-Audit/Audits/Cmp/Reports, accessed 26th April
2. Ethnic minorities dying of Covid-19 at higher rate, analysis shows. The Guardian, 22 April 2020. Available at: https://www.theguardian.com/world/2020/apr/22/racial-inequality-in-britain-found-a-risk-factor-for-covid-19
3. Cook T, Kursumovic E, Lennane S. Exclusive: deaths of NHS staff from covid-19 analysed. Health Serv J 2020 Apr 22. https://www.hsj.co.uk/exclusive-deaths-of-nhs-staff-fromcovid-19-analysed/7027471.article
4. Platt, L. and Warwick R. (2020) Are some ethnic groups more vulnerable to COVID-19 than others? Institute for Fiscal Studies ISBN 978-1-912805-75-4
5. Thebault R, Tran AB, Williams V. The coronavirus is infecting and killing black Americans at an alarmingly high rate. Washington Post 2020 Apr 7. https://www.washingtonpost.com/nation/2020/04/07/coronavirus-is-infecting-killing-black-americans-an-alarmingly-high-rate-post-analysis-shows
6. https://www.propublica.org/article/early-data-shows-african-americans-have-contracted-and-died-of-coronavirus-at-an-alarming-rate
7. https://www.ons.gov.uk/peoplepopulationandcommunity/healthandsocialcare/causesofdeath/datasets/deathregistrationsandoccurrencesbylocalauthorityandhealthboard, accessed 28th April
8. https://www.ons.gov.uk/peoplepopulationandcommunity/populationandmigration/populationestimates/datasets/populationestimatesforukenglandandwalesscotlandandnorthernireland
9. Wohland P, Burkitt M, Norman P, Rees P, Boden P and Durham H, ETHPOP Database, ESRC Follow on Fund "Ethnic group population trends". www.ethpop.org. Date of extraction 26th April 2020.
10. https://assets.publishing.service.gov.uk/government/uploads/system/uploads/attachment_data/file/835115/IoD2019_Statistical_Release.pdf
11. https://uk-air.defra.gov.uk/data/pcm-data#population_weighted_annual_mean_pm25_data
12. Bray I., Gibson A., White, J. Coronavirus disease 2019 mortality: a multivariate ecological analysis in relation to ethnicity, population density, obesity, deprivation and pollution. Public Health, 2020, 185, 261-263
13. Wu, X., Nethery, R., Sabath, B., Braun, D., & Dominici, F. (2020). Exposure to air pollution and COVID-19 mortality in the United States medRxiv 2020.04.05.20054502; doi: https://doi.org/10.1101/2020.04.05.20054502
14. ICNARC. ICNARC report on COVID-19 in critical care. ICNARC COVID-19 Study Case Mix Program Database 2020;:1–16.https://www.icnarc.org/Our-Audit/Audits/Cmp/Reports.
15. Nazroo J. Y. (2003). The structuring of ethnic inequalities in health: economic position, racial discrimination, and racism. American journal of public health, 93(2), 277–284. https://doi.org/10.2105/ajph.93.2.277
16. Bécares, L. (2015). Which ethnic groups have the poorest health?. In Jivraj, S. and Simpson, L. (eds.) Ethnic identity and inequalities in Britain. Bristol: The Policy Press
17. Wallace, S., Nazroo, J.Y. and Bécares, L. (2016)Cumulative exposure to racial discrimination across time and domains: exploring racism's long term impact on the mental health of ethnic minority people in the UK. American Journal of Public Health, 106(7), 1294-1300. doi: 10.2105/AJPH.2016.303121
18. Bos, V., Kunst, A., Keij-Deerenberg, I., Garssen, J., & Mackenbach, J. (2004). Ethnic inequalities in age- and cause-specific mortality in The Netherlands, International Journal of Epidemiology, 33(5), 1112–1119.
19. Bécares, L. and Nazroo, J. (2020). Racism is the root cause of ethnic inequities in COVID19, Discover Society, https://discoversociety.org/2020/04/17/racism-is-the-root-cause-of-ethnic-inequities-in-covid19/

For peer review only - http://bmjopen.bmj.com/site/about/guidelines.xhtml

20. Byrne, B., Alexander, C., Khan, O., Nazroo, J. And Shankley, W. (eds.) (2020) Ethnicity, Race and Inequality in the UK: State of the Nation, Bristol: Policy Press ISBN 978-1447351269)
21. Office for National Statistics. Coronavirus (COVID-19) related deaths by occupation, England and Wales deaths registered between 9 March and 25 May 2020. 2020. https://www.ons.gov.uk/peoplepopulationandcommunity/healthandsocialcare/causesofdeath/bulletins/coronaviruscovid19relateddeathsbyoccupationenglandandwales/deathsregisteredbetween9marchand25may2020
22. Nazroo, J.Y. (1997) The health of Britain's ethnic minorities: findings from a national survey, London: Policy Studies Institute ISBN 0 85374 709 1
23. Nazroo, J., Bhui, K. and Rhodes, J. (2020) 'Where next for understanding race/ethnic inequalities in severe mental illness? Structural, interpersonal and institutional racism', *Sociology of Health and Illness*, 42, 2, 262-276, doi: 10.1111/1467-9566.130012
24. Alexander, C. and Arday, J. (eds) (2015) Aiming Higher: Race Inequality and Diversity in the Academy.London: Runnymede Trust.
25. Zschirnt, E. and Ruedin, D. (2016): Ethnic discrimination in hiring decisions: a meta-analysis of correspondence tests 1990–2015. Journal of Ethnic and Migration Studies 42(7): 1115-34.
26. Ashe, S. and Nazroo, J. (2016) Equality, Diversity and Racism in the Workplace: A Qualitative Analysis of the 2015 Race at Work Survey, London: Business in The Community, https://race.bitc.org.uk/system/files/research/race_at_work_equality_diversity_and_racism_in_the_workplace_november_2016.pdf
27. Hogan, Bernie, and Brent Berry. 2011. "Racial and Ethnic Biases in Rental Housing: An Audit Study of Online Apartment Listings." City and Community 10 (4): 351–372.
28. Cohen, H. W., & Northridge, M. E. (2008). Getting political: racism and urban health. American journal of public health, 98(9 Suppl), S17–S19. https://doi.org/10.2105/ajph.98.supplement_1.s17
29. Bécares, L., Stafford, M., & Nazroo, J. (2009). Fear of racism, employment and expected organizational racism: their association with health. European Journal of Public Health, 19(5), 504-510.
30. Gee, G. C., Walsemann, K. M., & Brondolo, E. (2012). A life course perspective on how racism may be related to health inequities. American journal of public health, 102(5), 967–974. https://doi.org/10.2105/AJPH.2012.300666
31. Bécares, L., Nazroo, J., & Kelly, Y. (2015). A longitudinal examination of maternal, family, and area-level experiences of racism on children's socioemotional development: patterns and possible explanations. Social Science & Medicine, 142, 128-135
32. NIHR and UKRI Launch call for research on COVID-19 and ethnicity. Available at: https://www.nihr.ac.uk/news/nihr-and-ukri-launch-call-for-research-on-covid-19-and-ethnicity/24658
33. https://www.bmj.com/content/368/bmj.m268.full%22
34. Salway, S., Holman, D., Lee, C., McGowan, V., Ben-Shlomo, Y., Saxena, S. and Nazroo, J. (2020) 'Transforming the health system for the UK's multi-ethnic population', British Medical Journal, 368, m268 doi: 10.1136/bmj.m268
35. Nazroo, J. and Bécares, L. (2020) Racism is the key to understanding ethnic inequalities in COVID-19 – despite what UK government says, The Conversation, https://theconversation.com/racism-is-the-key-to-understanding-ethnic-inequalities-in-covid-19-despite-what-uk-government-says-148838
36. https://synergicollaborativecentre.co.uk/new-coronavirus-act-may-increase-the-mental-health-detention-of-ethnic-minority-people/%22

Acknowledgements
We are grateful for the support provided by Caelainn Barr, Editor, Data projects, Guardian News and

For peer review only - http://bmjopen.bmj.com/site/about/guidelines.xhtml

Media, and Niamh McIntyre, Niko Kommenda and Antonio Voce, Data projects, Guardian News and Media.

Funding

Nazroo's work on this paper was funded by the Economic and Social Research Council, grant number ES/R009341/1.

Data sharing

The analyses presented here make use of publicly available data. Links to the data are provided within the paper.

For peer review only

STROBE Statement—Checklist of items that should be included in reports of ***cross-sectional studies***

	Item No	Recommendation	Page No
**Title and abstract**	1	(*a*) Indicate the study's design with a commonly used term in the title or the abstract	1
		(*b*) Provide in the abstract an informative and balanced summary of what was done and what was found	2
**Introduction**			
Background/rationale	2	Explain the scientific background and rationale for the investigation being reported	3
Objectives	3	State specific objectives, including any prespecified hypotheses	3
**Methods**			
Study design	4	Present key elements of study design early in the paper	3-4
Setting	5	Describe the setting, locations, and relevant dates, including periods of recruitment, exposure, follow-up, and data collection	3-4
Participants	6	(*a*) Give the eligibility criteria, and the sources and methods of selection of participants	N/a
Variables	7	Clearly define all outcomes, exposures, predictors, potential confounders, and effect modifiers. Give diagnostic criteria, if applicable	4
Data sources/ measurement	8*	For each variable of interest, give sources of data and details of methods of assessment (measurement). Describe comparability of assessment methods if there is more than one group	4
Bias	9	Describe any efforts to address potential sources of bias	N/a
Study size	10	Explain how the study size was arrived at	N/a
Quantitative variables	11	Explain how quantitative variables were handled in the analyses. If applicable, describe which groupings were chosen and why	4
Statistical methods	12	(*a*) Describe all statistical methods, including those used to control for confounding	4
		(*b*) Describe any methods used to examine subgroups and interactions	4
		(*c*) Explain how missing data were addressed	N/a
		(*d*) If applicable, describe analytical methods taking account of sampling strategy	4
		(*e*) Describe any sensitivity analyses	N/a
**Results**			
Participants	13*	(a) Report numbers of individuals at each stage of study—eg numbers potentially eligible, examined for eligibility, confirmed eligible, included in the study, completing follow-up, and analysed	N/a
		(b) Give reasons for non-participation at each stage	N/a
		(c) Consider use of a flow diagram	N/a
Descriptive data	14*	(a) Give characteristics of study participants (eg demographic, clinical, social) and information on exposures and potential confounders	N/a
		(b) Indicate number of participants with missing data for each variable of interest	N/a
Outcome data	15*	Report numbers of outcome events or summary measures	4
Main results	16	(*a*) Give unadjusted estimates and, if applicable, confounder-adjusted estimates and their precision (eg, 95% confidence interval). Make clear which confounders were adjusted for and why they were included	5-6

For peer review only - http://bmjopen.bmj.com/site/about/guidelines.xhtml

		(*b*) Report category boundaries when continuous variables were categorized	N/a
		(*c*) If relevant, consider translating estimates of relative risk into absolute risk for a meaningful time period	N/a
Other analyses	17	Report other analyses done—eg analyses of subgroups and interactions, and sensitivity analyses	5-6
**Discussion**			
Key results	18	Summarise key results with reference to study objectives	6
Limitations	19	Discuss limitations of the study, taking into account sources of potential bias or imprecision. Discuss both direction and magnitude of any potential bias	6-7
Interpretation	20	Give a cautious overall interpretation of results considering objectives, limitations, multiplicity of analyses, results from similar studies, and other relevant evidence	6-8
Generalisability	21	Discuss the generalisability (external validity) of the study results	N/a
**Other information**			
Funding	22	Give the source of funding and the role of the funders for the present study and, if applicable, for the original study on which the present article is based	1

*Give information separately for exposed and unexposed groups.

Note: An Explanation and Elaboration article discusses each checklist item and gives methodological background and published examples of transparent reporting. The STROBE checklist is best used in conjunction with this article (freely available on the Web sites of PLoS Medicine at http://www.plosmedicine.org/, Annals of Internal Medicine at http://www.annals.org/, and Epidemiology at http://www.epidem.com/). Information on the STROBE Initiative is available at www.strobe-statement.org.

For peer review only
